# Supplementary material for: Association between oral microbiome and seven types of cancers in East Asian population: a two-sample Mendelian randomization analysis
Source: Front Mol Biosci. 2023 Nov 21;10:1327893. doi: 10.3389/fmolb.2023.1327893 (PMC10702768; doi:10.3389/fmolb.2023.1327893)
Supplement: Supplementary file 1 [file DataSheet1.ZIP › Supplementary Materials/MR plots of saliva/saliva═╝/colorectal cancer/pheno.598_to_colorectal cancer_forest.pdf]

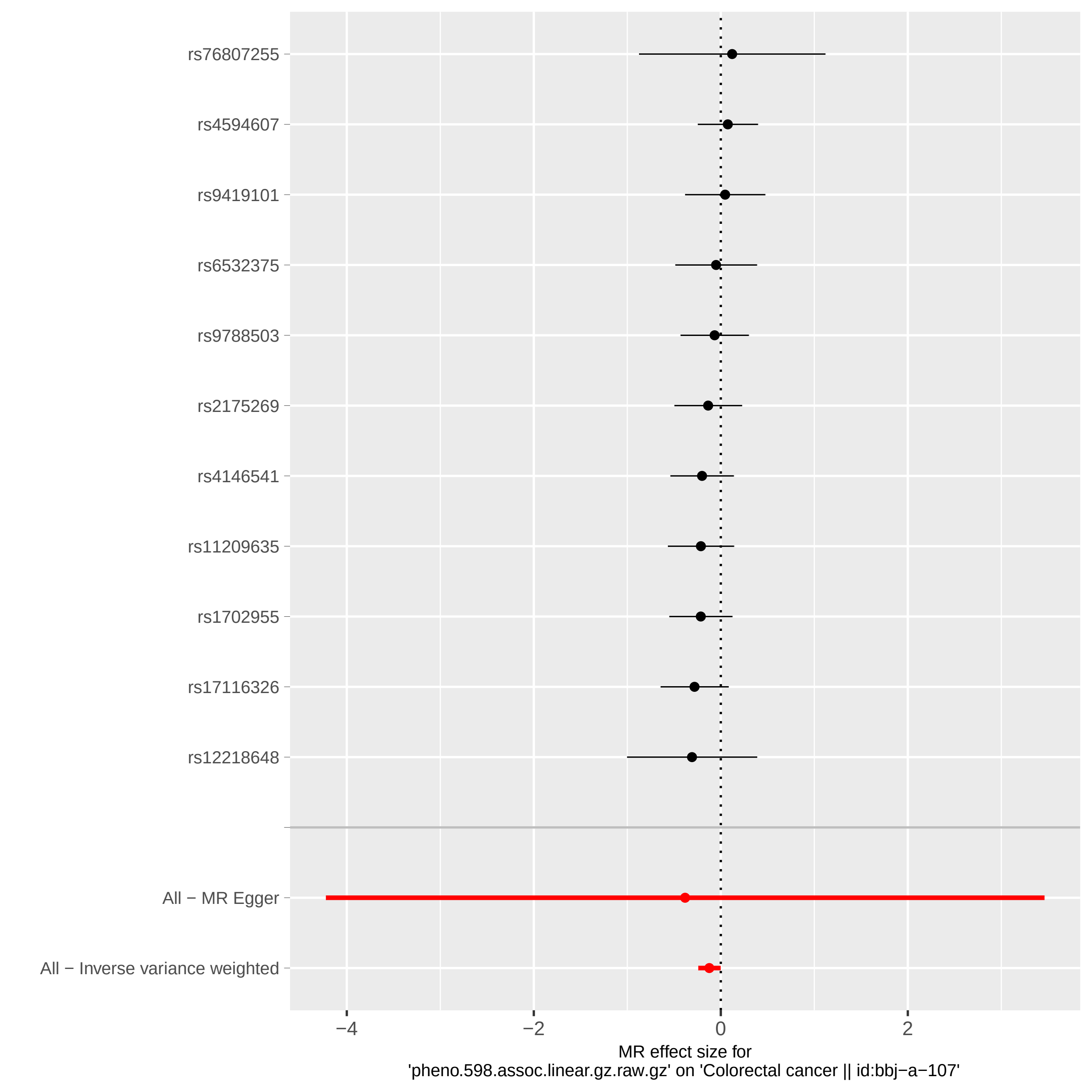
rs76807255
rs4594607
rs9419101
rs6532375
rs9788503
rs2175269
rs4146541
rs11209635
rs1702955
rs17116326
rs12218648
All − MR Egger
All − Inverse variance weighted
−4
−2
0
2
MR effect size for
'pheno.598.assoc.linear.gz.raw.gz' on 'Colorectal cancer || id:bbj−a−107'
